# Cigarette Smoke Exposure Attenuates T2R‐Mediated Apoptosis in Head and Neck Squamous Cell Carcinoma

**DOI:** 10.1002/hed.70290

**Published:** 2026-04-25

**Authors:** Kyle Polen, Sarah Sywanycz, Gavin Turner, Brianna L. Hill, Lily Huang, Zoey A. Miller, Casey Hanna, Lovely Raghav, Devraj Basu, Robert J. Lee, Ryan M. Carey

**Affiliations:** ^1^ Perelman School of Medicine University of Pennsylvania Philadelphia Pennsylvania USA; ^2^ Department of Otorhinolaryngology‐Head & Neck Surgery University of Pennsylvania Health System Philadelphia Pennsylvania USA; ^3^ Department of Physiology Perelman School of Medicine Philadelphia Pennsylvania USA

**Keywords:** apoptosis, bitter taste receptors (T2Rs), calcium signaling, cigarette smoke condensate (CSC), head and neck squamous cell carcinoma (HNSCC)

## Abstract

**Background:**

Tobacco use is associated with worse outcomes in head and neck squamous cell carcinoma (HNSCC). Bitter taste receptor (T2R) activation induces apoptosis via calcium‐dependent signaling, and higher T2R expression correlates with increased survival in HNSCC. However, the effects of cigarette smoke on T2R signaling remain unclear.

**Methods:**

HNSCC cell lines were treated with cigarette smoke condensate (CSC). T2R expression was measured by qPCR, and function evaluated by calcium imaging, viability, and apoptosis assays following stimulation with denatonium benzoate and flufenamic acid. T2R expression was also assessed in patient‐derived tumor tissue and The Cancer Genome Atlas (TCGA).

**Results:**

CSC reduced T2R expression, impaired calcium signaling, and diminished T2R‐mediated apoptosis, particularly in SCC47 cells. Lower expression was observed in CSC‐exposed patient‐derived tumor tissue and TCGA tumors from smokers versus nonsmokers.

**Conclusions:**

Cigarette smoke disrupts T2R signaling and reduces apoptosis in HNSCC, suggesting a mechanism by which ongoing tobacco use may alter tumor biology and treatment response.

## 1 Introduction

Head and neck squamous cell carcinomas (HNSCCs) are among the most common malignancies associated with tobacco use, a leading risk factor contributing to disease progression [1]. Primary treatments for HNSCC include surgery, radiation, and/or chemotherapy but often result in considerable morbidities impacting communication, swallowing, and cosmetic appearance [2]. Even with optimal treatment, the 5‐year survival rate for HNSCC is around 60%, and its incidence continues to rise predominantly due to the human papillomavirus (HPV) [3, 4, 5].

Despite the well‐documented link between smoking tobacco and the development of cancer, over 50% of patients continue to smoke after being diagnosed with HNSCC [6, 7], which leads to more aggressive disease, impaired locoregional control, and lower survival [8, 9, 10, 11, 12]. Beyond its established role in cancer progression, smoking has also been shown to impair bitter taste perception [13]. Research on bitter taste receptors (T2Rs) in HNSCC suggests that altered taste pathways may influence tumor biology, highlighting a potential link with smoking that warrants further investigation.

T2Rs, originally characterized for their role in bitter taste perception on the tongue, are now recognized as functional receptors expressed throughout the body in nongustatory tissues including cancer cells [14]. T2Rs are part of a large family of G‐protein coupled receptors (GPCRs) which includes approximately 25 different T2R isoforms encoded by *TAS2R* genes, and these receptors are activated by hundreds of bitter agonists [15, 16]. Canonical T2R signaling involves Gβγ‐activation leading to intracellular Ca^2+^ release from endoplasmic reticulum stores [17, 18, 19, 20]. Stimulation of endogenous T2Rs in HNSCC has been associated with Ca^2+^ influx into the mitochondria, leading to mitochondrial depolarization, reactive oxygen species (ROS) production, and caspase‐3/7‐mediated apoptosis [21, 22]. Clinical data from The Cancer Genome Atlas (TCGA) suggest higher expression of *TAS2R4* and *TAS2R14* within HNSCC tumor samples is correlated with improved patient survival [21, 23, 24], and *TAS2R14* expression is increased in HPV‐positive (HPV+) tumors compared to HPV‐negative (HPV−) [25]. Lidocaine, an agonist of T2R14, triggers HNSCC apoptosis via T2R‐mediated pathways [25], prompting the initiation of a phase I clinical trial evaluating lidocaine in the neoadjuvant setting for HNSCC [26]. Many other cancers, including breast and thyroid cancers, also express T2Rs [25, 27, 28], suggesting these receptors may play a broader role in cancer physiology and have prognostic or therapeutic value. In fact, a large randomized controlled clinical trial showed that peritumoral injection of T2R14 agonist lidocaine prior to breast cancer surgery significantly improved patient survival compared to no injection [29, 30].

While T2Rs hold potential for innovative cancer therapies, cigarette smoke exposure may pose a challenge to their efficacy or alter endogenous T2R biology. Studies have indicated that cigarette smoke condensate (CSC) alters airway epithelial gene expression and function in as little as 24 h [31, 32], and it is known that individuals who smoke cigarettes have diminished bitter taste [13]. Taken together, we hypothesized that exposure to chemicals found in cigarette smoke in the form of CSC may attenuate the expression and functional responses of T2Rs in HNSCC cells, diminishing the effectiveness of T2R‐mediated apoptosis.

## 2 Materials and Methods

### 2.1 Cell Culture

SCC47 (UM‐SCC‐47; Millipore SCC071), FaDu (ATCC HTB‐43), and RPMI2650 (ATCC CCL‐30) cells were obtained from MilliporeSigma (St. Louis, MO USA) or ATCC (Manassas, VA, USA). All cancer cell lines were cultured in high‐glucose Dulbecco's Modified Eagle Medium (DMEM; Corning; Glendale, AZ, USA), combined with 10% FBS (Genesee Scientific; El Cajon, CA, USA), a 1% penicillin/streptomycin mixture (Gibco; Gaithersburg, MD, USA), and 1% nonessential amino acids (NEAA; Gibco). Utilized cell lines were below 25 passages, and cells were used in experiments at 70%–80% culture confluency.

### 2.2 Chemicals

CSC 40 mg/mL stock dissolved in 100% dimethyl sulfoxide (DMSO) was obtained from Murty Pharmaceuticals Inc. (Lexington, KY) and diluted using sterile Phenol‐free DMEM supplemented with 10% FBS, 1% penicillin/streptomycin, and 1% NEAA.

The T2R agonists denatonium benzoate (agonist for T2R4, 8, 10, 13, 39, 43, 46, and 47) [21] and flufenamic acid (agonist for T2R14) were used for experimental work (MilliporeSigma) [25, 33]. Hank's Balanced Salt Solution (HBSS) buffered with 20 mM HEPES (pH 7.4) was used for diluting these agonists to desired concentrations.

### 2.3 Quantitative Reverse Transcription PCR

Cultured cells were resuspended in TRIzol (Thermo Fisher Scientific). RNA was extracted using a Direct‐zol RNA Kit (Zymo Research) and reverse transcribed using the High‐Capacity cDNA Reverse Transcription Kit (Thermo Fisher Scientific). Quantitative reverse transcription PCR (qPCR) was performed per the manufacturer's protocol using Taqman qPCR probes for *TAS2R4* and *TAS2R14* genes and housekeeping gene UBC (QuantStudio 5; Thermo Fisher Scientific), which was utilized as an endogenous control [34].

### 2.4 Live Cell Imaging

Cells were loaded with 5 μM of Fluo‐4‐AM (Thermo Fisher Scientific) for 45 min in the dark at room temperature. Hank's Balanced Salt Solution (HBSS) buffered with 20 mM HEPES (pH 7.4) was used during imaging. Nikon Eclipse TS100 (20 × 0.75 NA PlanApo objective), FITC filters (Chroma Technologies), QImaging Retiga R1 camera (Teledyne; Tucson, AZ, USA), MicroManager, and Xcite 120 LED Boost (Excelitas Technologies; Mississauga, Canada) were used to image cells at ×20. Quantitative fluorescence values were measured using ImageJ.

### 2.5 Apoptosis and Viability

After incubating cells in media treated with CSC (“smoked”) or non‐CSC media (“control”), fresh Phenol‐free DMEM supplemented with 10% FBS, 1% penicillin/streptomycin, and 1% NEAA was added with CellEvent Caspase‐3/7 dye in combination with bitter agonists. Cells were imaged for 15 h using the same equipment listed above. Fluorescence was measured at 495 nm excitation and 540 nm emission, with quantitative fluorescence values measured using ImageJ.

To assess cell viability, crystal violet (0.1% in deionized water with 10% acetic acid) was used to stain viable cells following exposure to compounds of interest. The stains were washed with deionized water, left to dry for 12 h, then dissolved with 30% acetic acid in deionized water. Absorbance was measured at 590 nm on a Tecan Spark 10 M.

### 2.6 Western Blot Analysis

SCC47 cells were plated in triplicate and cultured in media with or without 25 μg/mL CSC for 48 h. Protein was extracted using RIPA lysis buffer supplemented with complete EDTA‐free protease inhibitor cocktail (MilliporeSigma). Protein content was quantified using DC Protein Assay (Bio‐Rad). Equal amounts of protein were separated on NuPAGE 4%–12% Bis‐Tris gels and transferred to nitrocellulose membranes. Membranes were blocked with 5% nonfat dry milk in TBST and incubated overnight at 4°C with primary antibodies against caspase‐3 (1:1000; RRID:AB_2798429) or β‐actin (1:1000; RRID:AB_2242334). HRP‐conjugated secondary antibodies (goat anti‐rabbit IgG‐HRP, RRID:AB_631746; goat anti‐mouse IgG‐HRP, RRID:AB_3717730) were incubated at 1:1000 for 1 h at room temperature. Blots were developed using SuperSignal PLUS Chemiluminescent Substrate (Thermo Fisher Scientific) and imaged on a ChemiDoc MP system (Bio‐Rad).

### 2.7 Patient‐Derived Tumor Specimens

Patient‐derived tissue was collected with approval from the Institutional Review Board of the University of Pennsylvania (IRB #417200) to generate HPV+ oropharyngeal squamous cell carcinoma (OPSCC) tumor slices, as previously described [35]. Tumor tissue was sectioned into 300‐μm‐thick slices using a vibratome (Precisionary Compresstome Vibratome VF‐510‐0Z; oscillations, 6; speed, 3) and plated with 3 independent slices per well across 6 wells per condition. Slices were cultured in media treated with 25 μg/mL CSC or untreated media for 48 h. RNA was extracted and assessed for quality; samples with low concentration or poor 260/230 ratios were excluded. qPCR was performed in duplicate per the protocols described above.

### 2.8 The Cancer Genome Atlas Analysis

RNA‐seq and corresponding clinical data from The Cancer Genome Atlas (TCGA) were downloaded from the Genomic Data Commons (GDC) portal and processed using the nf‐core/rnaseq pipeline (STAR alignment followed by Salmon quantification and DESeq2 for normalization; https://github.com/nf‐core/rnaseq). HPV+ HNSCC samples were identified based on clinical annotation and the presence of > 1000 reads aligning to the HPV E6 and E7 genes, as previously described [36]. Samples were filtered by oropharyngeal subsite, and smoking status was obtained from GDC clinical annotations classifying patients as ever smoker or never smoker. One sample without available smoking data was excluded from analysis. Log‐transformed counts per million values for *TAS2R4* and *TAS2R14* were compared between groups using Welch's *t*‐test.

### 2.9 Data Analysis and Statistics

Statistical analysis was conducted using GraphPad Prism (San Diego, CA, USA). For two‐group comparisons, unpaired *T*‐tests were applied. When comparing more than two groups, one‐way ANOVA was utilized. All figures are annotated with *p* < 0.05 (*), *p* < 0.01 (**), *p* < 0.001 (***), *p* < 0.0001 (****), and error bars represent mean ± SEM.

## 3 Results

### 3.1 Baseline Effect of Cigarette Smoke Condensate (CSC) on HNSCC Cells

**FIGURE 1:**
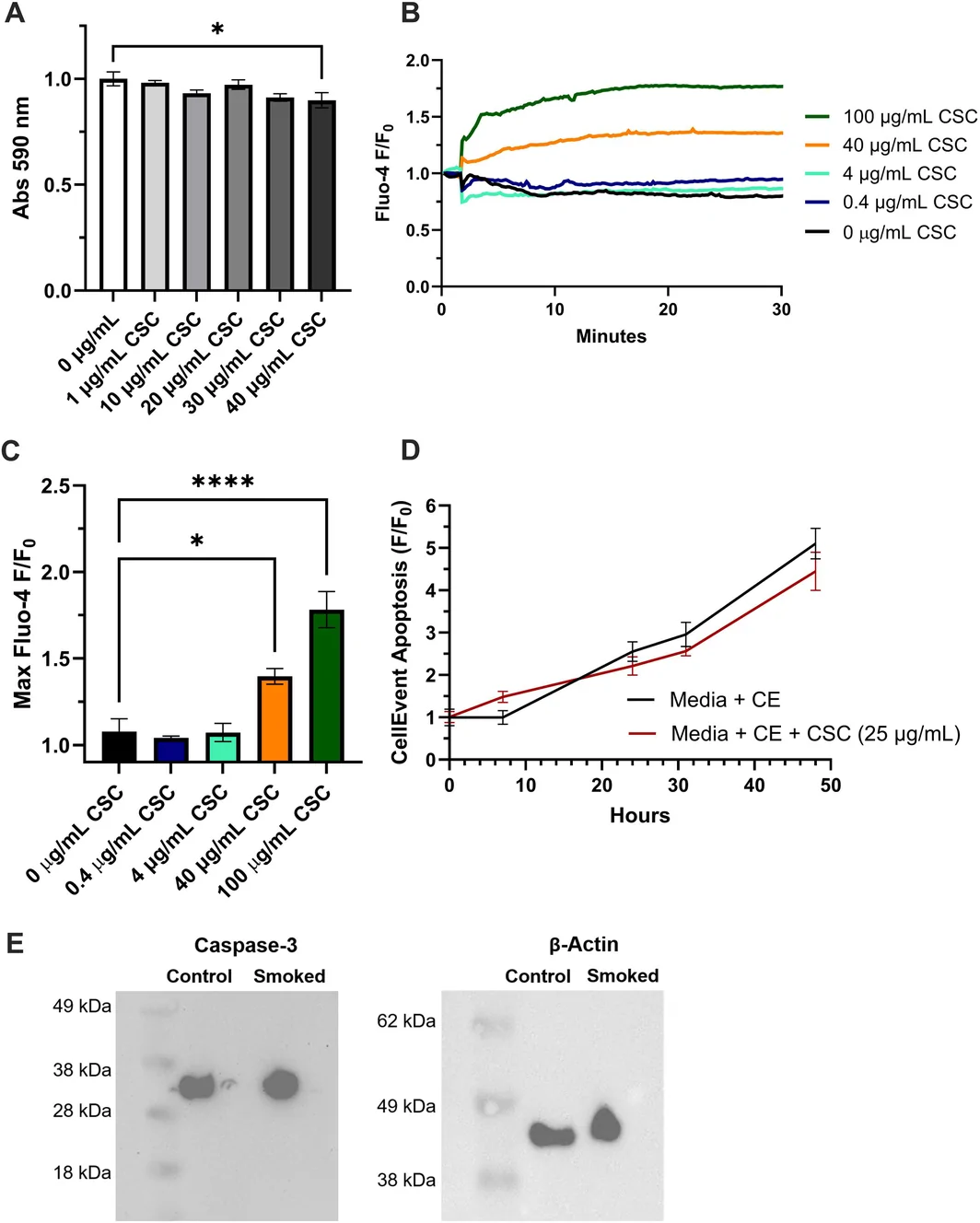
Baseline effect of cigarette smoke condensate on HNSCC cell viability, calcium release, and apoptosis. SCC47 cells were exposed to increasing concentrations of cigarette smoke condensate (CSC; 0–40 μg/mL) for 24 h. Cell viability was then measured using crystal violet staining and absorbance quantified at 590 nm (A). SCC47 cells were also acutely exposed to increasing concentrations of CSC (0.4 μg/mL to 100 μg/mL in HBSS) with intracellular calcium levels measured using Fluo‐4 dye over 30 min. Real‐time fluorescence changes over time are shown in (B) as representative traces (Fluo‐4 *F*/*F*₀), while (C) presents maximum fluorescence values. SCC47 cells were cultured in media with or without 25 μg/mL CSC for 48 h, and caspase‐3/7 cleavage was measured using CellEvent dye (CE) over that time period (*n* = 6). Data are presented as fluorescence intensity captured at four time points, with each normalized to fluorescence at time point zero (*F*/*F*₀) (D). Western blot analysis for caspase‐3 was performed on SCC47 cells after 48‐h culture with (“smoked”) or without 25 μg/mL CSC (“control”), with β‐Actin as loading control (E). Full‐length caspase‐3 is approximately 32–35 kDa; cleaved protein would be present at 17–19 kDa. Data are presented as mean ± SEM; significance by one‐way ANOVA *p* < 0.05 (*), *p* < 0.0001 (****). [Color figure can be viewed at wileyonlinelibrary.com]

Cell viability in the presence of CSC was examined by treating SCC47 (HPV+ oral cancer) cells with 0, 1, 10, 20, 30, or 40 μg/mL of CSC for 24 h. It was observed that cell viability was only minimally affected at 40 μg/mL with no significant effects observed at 30 μg or lower (Figure 1A). It was also confirmed that 40 μg/mL is approximately the threshold at which CSC itself acutely mobilizes Ca^2+^ in SCC47 cells (Figure 1B,C). To remain below these thresholds and consistent with previous work [32, 37], a dose of 25 μg/mL was used for pretreatment in media containing FBS. To confirm that the working dose of CSC did not independently induce apoptosis, we verified that caspase‐3/7 cleavage was similar in cells cultured in CSC media and control media over 48 h via CellEvent fluorescence (Figure 1D). Western blot analysis further confirmed the absence of caspase‐3 cleavage in both conditions (Figure 1E).

### 3.2 Cigarette Smoke Exposure Decreases Expression of TAS2R4 and TAS2R14 in HNSCC

**FIGURE 2:**
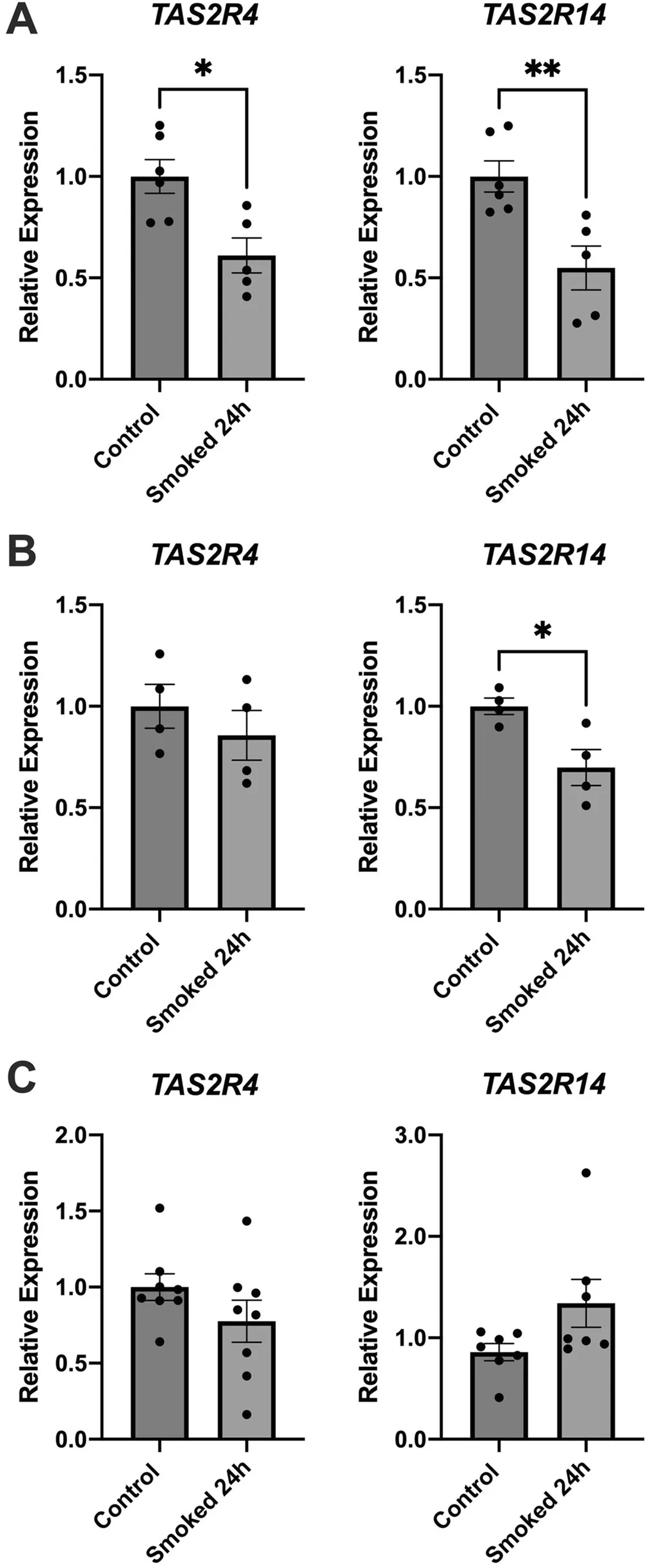
Effect of cigarette smoke condensate exposure on *TAS2R4* and *TAS2R14* expression in HNSCC cell lines. Quantitative RT‐PCR analysis of *TAS2R4* and *TAS2R14* expression in SCC47 (A), FaDu (B), and RPMI 2650 (C) cells after 24‐h exposure to 25 μg/mL cigarette smoke condensate‐treated media (“smoked”) or untreated media (“control”). Expression is relative to the housekeeping gene UBC. Data represent individual data points and mean ± SEM; significance by *T*‐test *p* < 0.05 (*), *p* < 0.01 (**).

Experiments utilized HNSCC cell lines SCC47 (HPV+ oral cancer), FaDu (HPV− hypopharyngeal cancer), and RPMI 2650 (HPV− nasal cavity cancer), which each exhibit varying endogenous expression levels of genes encoding the ~25 members of the T2R receptor family [21]. *TAS2R4* and *TAS2R14* expression have previously been found to correlate with survival in patients diagnosed with HNSCC, and they encode T2Rs which regulate cancer cell apoptosis in vitro [21, 25, 38]. To understand if CSC exposure can alter the expression of *TAS2R4* and *TAS2R14*, cells were incubated for 24 h in the presence or absence of 25 μg/mL CSC (abbreviated “smoked” vs. “control”, respectively). qPCR showed significantly decreased expression of both *TAS2R4* and *TAS2R14* in smoked SCC47 cells compared to nonsmoked cells (Figure 2A). In smoked FaDu cells, a similar decrease in *TAS2R14* expression was observed (Figure 2B). In RPMI cells, statistically significant changes in expression were not observed (Figure 2C). These data in SCC47 and FaDu cells suggest that CSC may differentially alter the gene expression of two abundant T2Rs involved in HNSCC apoptosis, *TAS2R4* and *TAS2R14*.

### 3.3 Cigarette Smoke Reduces T2R Functionality in HNSCC

It is well established that activation of T2Rs leads to an elevated intracellular Ca^2+^ response in various cell types [22]. To examine if T2R functionality in HNSCC cells is altered by CSC, Ca^2+^ responses in cells pre‐treated with and without CSC were recorded in the presence of bitter agonists via the Ca^2+^ indicator dye Fluo‐4‐AM. Denatonium benzoate, widely recognized as one of the most bitter‐tasting substances [39], was utilized for its activation of many T2Rs including T2R4 (15 mM; agonist for T2R4, 8, 10, 13, 39, 43, 46, and 47) [21]. Flufenamic acid (FFA) was used for its selective activation of T2R14 (500 μM) [25, 33]. In HNSCC cell lines, these bitter agonists increase intracellular Ca^2+^ levels through the activation of T2R4‐ and T2R14‐mediated G protein signaling, resulting in endoplasmic reticulum IP_3_ receptor‐dependent Ca^2+^ release [22, 40]. Measuring Ca^2+^ levels in cells exposed to bitter agonists is thus generally accepted as a method for assessing T2R function [21, 25].

**FIGURE 3:**
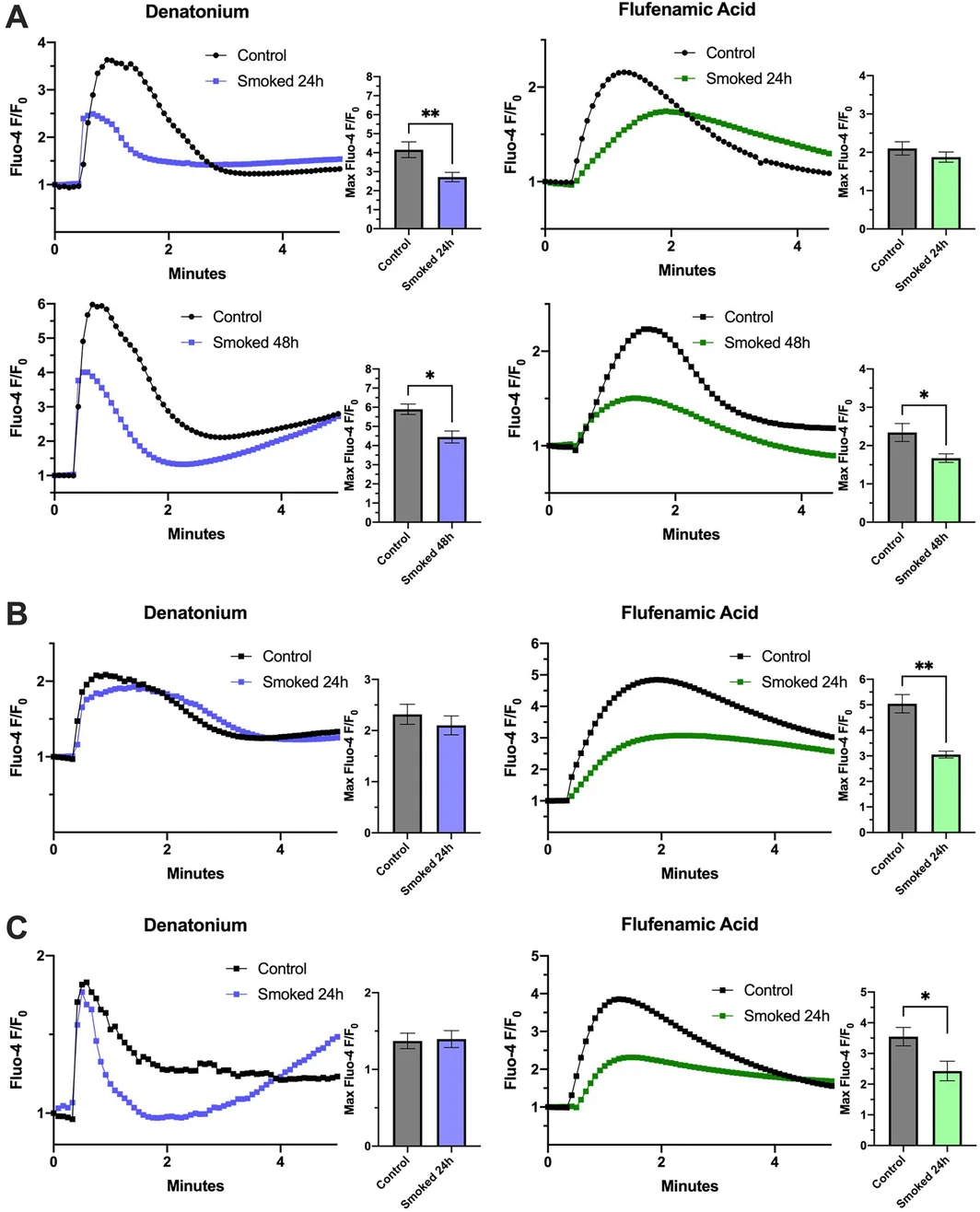
Cigarette smoke condensate exposure impacts T2R‐mediated Ca^2+^ responses in HNSCC cell lines. Intracellular Ca^2+^ responses were measured using Fluo‐4‐AM dye in SCC47 cells treated with or without cigarette smoke condensate (CSC; “smoked” vs. “control”) for 24 h (*n* = 8) or 48 h (*n* = 4) then stimulated with bitter agonists denatonium benzoate (15 mM, purple) or flufenamic acid (500 μM, green) suspended in HBSS (A). The same protocol was repeated for FaDu cells (B, *n* = 4) and RPMI cells (C, *n* = 8) treated with or without CSC for 24 h. Representative traces of real‐time fluorescence are shown with corresponding bar graphs of maximum fluorescence intensity. Data are presented as mean ± SEM; significance by T‐test *p* < 0.05 (*), *p* < 0.01 (**). [Color figure can be viewed at wileyonlinelibrary.com]

In SCC47 cells exposed to CSC for 24 and 48 h, Ca^2+^ responses stimulated by denatonium benzoate were significantly lower compared to nonexposed cells. This may suggest a reduction in T2R4 activity. Flufenamic acid‐stimulated Ca^2+^ release was not significantly different in cells smoked for 24 h, but when smoked for 48 h, a significant reduction in Ca^2+^ release was observed (Figure 3A). Smoked FaDu and RPMI cells also exhibited a significantly lower Ca^2+^ response at 24 h (Figure 3B,C). These data may suggest T2R4 and T2R14 functionality is reduced in certain cell lines exposed to CSC, with the most pronounced effects seen in SCC47.

### 3.4 Cigarette Smoke Diminishes T2R‐Mediated HNSCC Apoptosis

Because bitter agonists induce apoptosis in HNSCC, we investigated how pre‐exposure to CSC alters this response. CellEvent was used to measure caspase‐3 and caspase‐7 cleavage when cells were exposed to T2R agonist denatonium benzoate. More fluorescence indicates higher levels of caspase‐3/7 cleavage and therefore more apoptosis [41].

**FIGURE 4:**
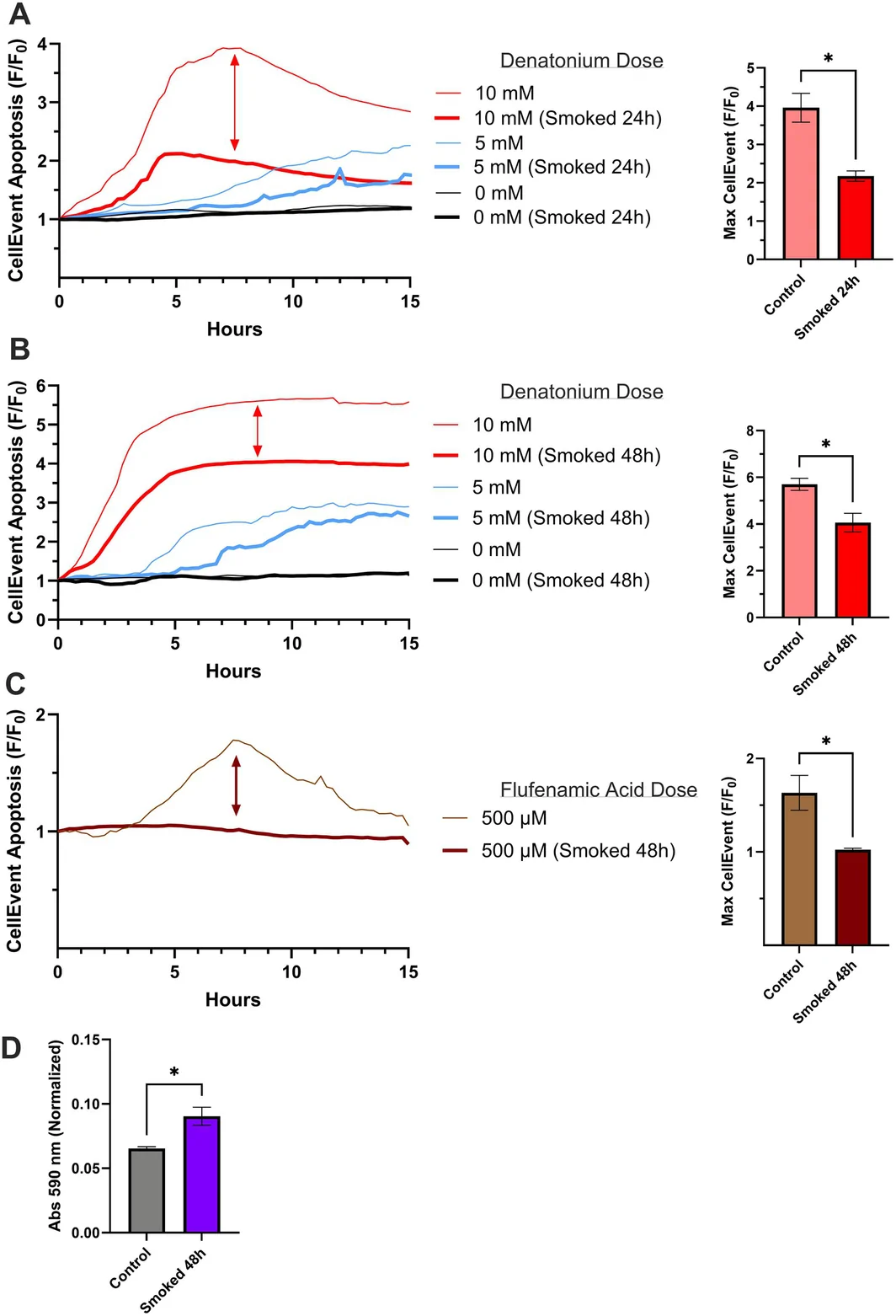
Apoptotic changes in SCC47 cells treated with cigarette smoke condensate before stimulation with bitter agonists. SCC47 cells were incubated in cigarette smoke condensate‐treated media (“smoked”) or untreated media (“control”) for 24 h, followed by the addition of CellEvent Caspase‐3/7 dye in media and denatonium benzoate at 5 mM (blue) and 10 mM (red). The y‐axis represents CellEvent fluorescence (*F*/*F*₀), a marker of cell apoptosis normalized to time point zero. Cells were imaged over 15 h, and fluorescence was measured at 495 nm excitation and 540 nm emission. A corresponding bar graph represents the peak fluorescence directly comparing smoked (red) to nonsmoked (pink) cells when exposed to 10 mM of denatonium (A, *n* = 3). Cells were also incubated in CSC‐treated media for 48 h then exposed to 15 h of denatonium benzoate (B, *n* = 3). Additionally, cells were exposed to 15 h of flufenamic acid at a dose of 500 μM (C, *n* = 3). Corresponding bar graphs show the peak fluorescence over the experimental time frame. Using crystal violet dye and the same pre‐treatment protocol with CSC for 48 h, SCC47 cells were stained after exposure to 10 mM denatonium benzoate for 6 h. Cell viability was measured at 590 nm with absorbance normalized to a corresponding well plated without bitter agonist (D, *n* = 3). With crystal violet, greater fluorescence correlates with a greater number of viable cancer cells. Data represent mean ± SEM; significance by *T*‐test *p* < 0.05 (*). [Color figure can be viewed at wileyonlinelibrary.com]

In comparing 24‐h smoked and nonsmoked SCC47 cells, it was found that smoked cells experienced significantly less apoptosis when exposed to both 10 and 5 mM denatonium benzoate (Figure 4A). The observed trend also suggests a potential dose‐related effect that warrants further investigation. Similarly, at a 48‐h timepoint, smoked SCC47 cells experienced significantly less denatonium benzoate‐mediated apoptosis compared to nonsmoked controls (Figure 4B). When cell death was stimulated using 500 μM flufenamic acid, 48‐h smoked cells experienced significantly less apoptosis compared to nonsmoked controls, suggesting T2R14 signaling is also diminished (Figure 4C). To validate the observed changes in the apoptosis assay, cell viability was also assessed using crystal violet staining. After 6 h of exposure to 10 mM denatonium benzoate, smoked SCC47 cells demonstrated significantly higher viability compared to nonsmoked cells (Figure 4D). Together, these data suggest that smoke exposure may reduce susceptibility to T2R‐mediated effects on cell viability and apoptosis.

### 3.5 TAS2R Expression Is Reduced in Patient‐Derived Tumor Tissue and Tumors From Smokers

**FIGURE 5:**
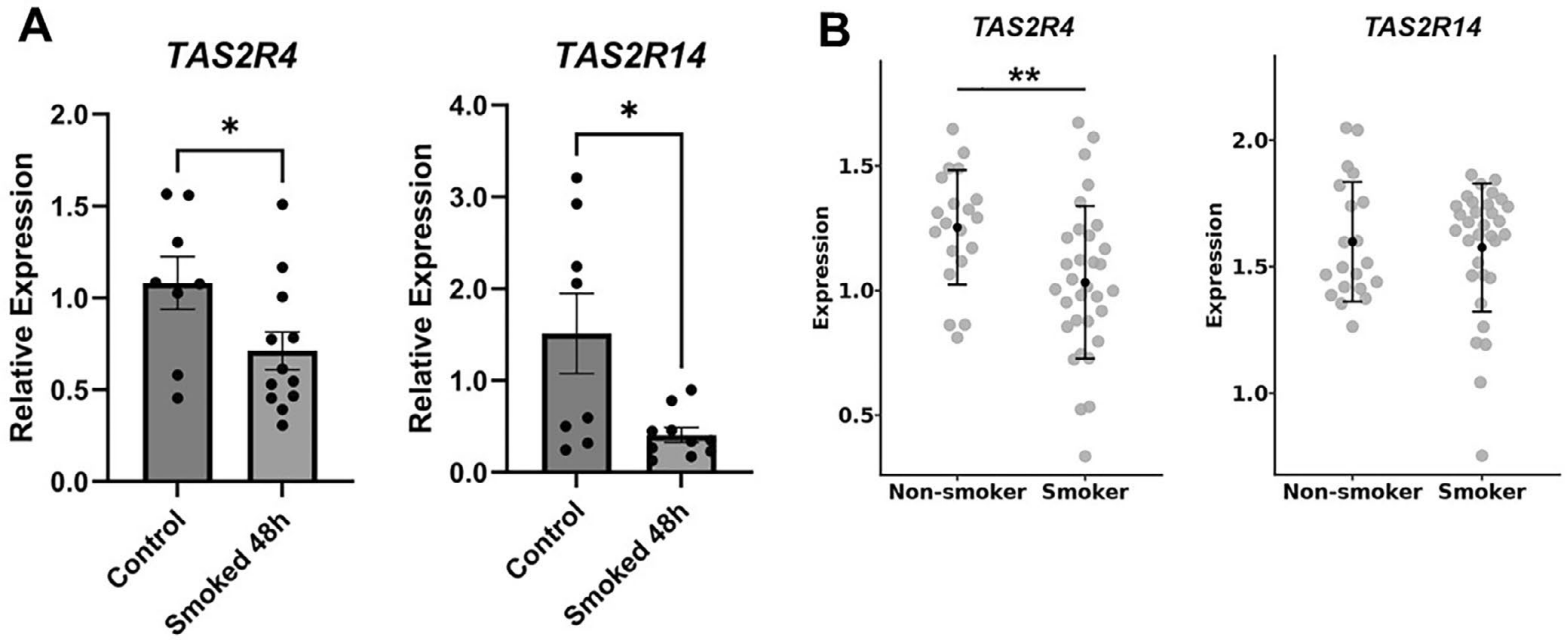
*TAS2R* expression in patient‐derived tumor tissue and TCGA tumors. Quantitative RT‐PCR analysis of *TAS2R4* and *TAS2R14* expression in tumor slices from HPV+ tonsillar SCC cultured in 25 μg/mL cigarette smoke condensate‐treated media (“smoked 48 h”) or untreated media (“control”). Expression is relative to the housekeeping gene UBC (A). RNA‐seq expression (log‐transformed counts per million) of *TAS2R4* and *TAS2R14* in HPV+ oropharyngeal squamous cell carcinoma tumors from The Cancer Genome Atlas, stratified by smoking status (nonsmoker, *n* = 32; smoker, *n* = 20) (B). Data represent individual data points with mean ± SEM; significance by *T*‐test *p* < 0.05 (*), *p* < 0.01 (**).

Patient‐derived tumor slices demonstrated decreased *TAS2R4* and *TAS2R14* expression when cultured in CSC compared to controls (Figure 5A). To examine whether smoking status is associated with TAS2R expression in a larger patient cohort, RNA‐seq data from 52 HPV+ OPSCC tumors in TCGA were analyzed by smoking status (nonsmoker, *n* = 32; smoker, *n* = 20). *TAS2R4* expression was found to be lower in tumors from smokers compared to nonsmokers (Figure 5B).

## 4 Discussion

Despite advances in treatment, HNSCC remains a disease with limited effective molecular therapies and significant morbidity from standard interventions such as surgery, radiation, and chemotherapy [42]. This reflects the need for novel therapeutic approaches. T2Rs have emerged as intriguing targets given their enriched expression in HNSCC and their ability to induce apoptosis via Ca^2+^‐dependent pathways [21]. Importantly, many bitter compounds—including FDA‐approved drugs [43], plant compounds [44, 45], and microbial products [46, 47, 48]—are known T2R agonists, suggesting potential for therapeutic exploitation.

In this study, we demonstrate that CSC suppresses T2R expression and impairs T2R‐mediated signaling and apoptosis in HNSCC cells. These effects were most pronounced in SCC47 cells, where both *TAS2R4* and *TAS2R14* expression were reduced following CSC exposure. In FaDu cells, *TAS2R14* downregulation was also observed. These transcriptional changes corresponded with diminished Ca^2+^ flux and blunted apoptotic responses to bitter agonists denatonium benzoate and flufenamic acid, indicating functional disruption of T2R signaling.

Interestingly, denatonium—an agonist of multiple T2Rs including T2R4—elicited more dramatic differences in apoptosis between CSC‐treated and untreated cells than flufenamic acid, which primarily targets T2R14. These findings suggest that CSC may interfere with broader T2R networks and not just individual receptor pathways. The observed differences in T2R expression and response between cell lines may reflect underlying biological variations that warrant further exploration in studies dedicated to this aim.

The in vitro findings were supported by data from patient‐derived tissue and TCGA analyses. Ex vivo tumor slices from HPV+ tonsillar SCC demonstrated reduced *TAS2R4* and *TAS2R14* expression following CSC exposure, consistent with results seen in the established cell lines. Additionally, analysis of TCGA RNA‐seq data from 52 OPSCC tumors revealed significantly lower *TAS2R4* expression in tumors from smokers compared to nonsmokers. This TCGA analysis is limited by the fact that smoking history lacks details regarding active smoking at the time of diagnosis and treatment. Nevertheless, these data provide supporting evidence that cigarette smoke exposure may reduce T2R gene expression in human HNSCC tumors.

Our results suggest a potential mechanism by which cigarette smoking contributes to cancer progression: by dampening T2R signaling, cigarette smoke may render tumor cells less responsive to bitter ligands that normally induce apoptosis. This aligns with clinical observations linking continued smoking during treatment to worse outcomes including impaired tumor control and metastasis [8, 9]. One possible explanation is that cigarette smoke triggers a broader adaptive response in tumor cells—analogous to its known induction of ABC transporters like ABCG2 to enhance efflux of smoke‐related toxins [49]—that includes downregulation of T2Rs to limit pro‐apoptotic signaling. This effect may not only impact T2R‐targeted therapies under investigation such as lidocaine [26], but could also broadly affect cancer cell regulation, as endogenous bitter ligands and microbiome constituents such as 3‐oxo‐C12SHL play integral roles in immune signaling and regulate the tumor microenvironment [46, 47, 48].

Another area warranting investigation is the interaction between T2Rs and emerging alternative tobacco products, such as electronic cigarettes, vaporizers, and nicotine pouches. Although conventional cigarettes remain the most widely used tobacco product among adults in the United States, the increasing popularity of these next‐generation products deemed to be less harmful still introduces novel chemical constituents to the microenvironment, including the bitter molecule nicotine [50, 51, 52, 53]. The molecular composition of cigarette alternatives, including nicotine salts, flavoring agents, and other additives, may differentially influence T2R expression and function compared to traditional tobacco smoke. Examining how these substances interact with T2Rs could provide insights into their potential effects on the pathophysiology of HNSCC. Future research is required to characterize the specific components of cigarette smoke and alternative tobacco products that may modulate T2R signaling pathways, which could have implications for both tumor profiling and therapeutic interventions.

Several limitations in our study should be acknowledged. We primarily relied on in vitro models which do not fully recapitulate the complex in vivo tumor microenvironment. The CSC doses used were also much lower than the estimated exposures from smoking, as one cigarette is believed to expose the human airway to over 10 000 μg of particulate matter [54]. However, the human airway does contain many more cells capable of metabolizing these particles and has metabolic, vascular, and immune components that may reduce the effective dose delivered to individual cancer cells. It is difficult to fully compare our exposure here to in vivo exposures, except to say that our in vitro exposure is likely much lower than what a frequent smoker would incur. Our study was also limited to a small number of cell lines with effects most consistently observed in SCC47 cells; the inherent variability in T2R expression across tumors may somewhat limit generalizability. Future expanded studies in more types of cell lines, larger patient‐derived cohorts, and animal models are needed to validate these findings and determine their translational relevance.

## 5 Conclusion

Our study reveals a previously uncharacterized impact of CSC exposure on T2R function in HNSCC cells, leading to diminished *TAS2R4* and *TAS2R14* gene expression and blunted T2R‐mediated Ca^2+^ signaling and apoptosis. These findings suggest that cigarette smoke exposure may alter tumor cell responses to T2R agonists, with potential implications for T2R‐targeted therapeutic approaches or responsiveness to endogenous T2R ligands. Future exploration of T2R signaling effects across cancer types and preclinical models, particularly in the context of tobacco exposure, could inform new approaches for biomarker development or therapeutic strategies targeting bitter pathways in smoking‐related malignancies.

## Funding

This work was supported by National Institutes of Health (HL168060, AI167971, DE034474, UH2CA267502, and R01DE034056).

## Conflicts of Interest

The authors declare no conflicts of interest.

## Data Availability

The data that support the findings of this study are available from the corresponding author upon reasonable request.
